# The Different Gene Expression Profile in the Eutopic and Ectopic Endometrium Sheds New Light on the Endometrial Seed in Endometriosis

**DOI:** 10.3390/biomedicines12061276

**Published:** 2024-06-08

**Authors:** Muhammad Assad Riaz, Ezekiel Onyonka Mecha, Charles O. A. Omwandho, Felix Zeppernick, Ivo Meinhold-Heerlein, Lutz Konrad

**Affiliations:** 1 Department of Gynecology and Obstetrics, University of Giessen, 35392 Giessen, Germany; muhammad.a.riaz@gyn.med.uni-giessen.de (M.A.R.); felix.zeppernick@gyn.med.uni-giessen.de (F.Z.); ivo.meinhold-heerlein@gyn.med.uni-giessen.de (I.M.-H.); 2 Department of Biochemistry, University of Nairobi, Nairobi 00100, Kenya; emecha@uonbi.ac.ke; 3 Department of Health Sciences, Kirinyaga University, Kirinyaga 10300, Kenya; omwandho@kyu.ac.ke

**Keywords:** endometrium, endometriosis, epithelial–mesenchymal transition, EMT, claudins, keratins, seed and soil

## Abstract

The changes in endometrial cells, both in the eutopic endometrium of patients with and without endometriosis and in lesions at ectopic sites, are frequently described and often compared to tumorigenesis. In tumorigenesis, the concept of “seed and soil” is well established. The seed refers to tumor cells with metastatic potential, and the soil is any organ or tissue that provides a suitable environment for the seed to grow. In this systematic review (PRISMA-S), we specifically compared the development of endometriosis with the “seed and soil” hypothesis. To determine changes in the endometrial seed, we re-analyzed the mRNA expression data of the eutopic and ectopic endometrium, paying special attention to the epithelial–mesenchymal transition (EMT). We found that the similarity between eutopic endometrium without and with endometriosis is extremely high (~99.1%). In contrast, the eutopic endometrium of patients with endometriosis has a similarity of only 95.3% with the ectopic endometrium. An analysis of EMT-associated genes revealed only minor differences in the mRNA expression levels of claudin family members without the loss of other cell–cell junctions that are critical for the epithelial phenotype. The array data suggest that the changes in the eutopic endometrium (=seed) are quite subtle at the beginning of the disease and that most of the differences occur after implantation into ectopic locations (=soil).

## 1. Introduction

The histologic appearance of endometrial glands and stroma outside the uterine cavity is the definition of endometriosis used by pathologists worldwide [1]. In contrast to epithelial endometriosis, which is quite rare, a large study of pelvic endometriosis found a higher percentage of cases (123/274 = 44.9%) with stromal endometriosis [2]. Recently, we provided strong evidence that stromal endometriosis is also common (~53%) in catamenial pneumothorax when caused by ectopic endometrial lesions [3]; however, the incidence of catamenial pneumothorax is rather low (0.2–1.5%). In many patients, endometriosis causes pain and/or infertility [4].

The histologic definition of endometriosis was recently described as outdated and does not reflect the true manifestations of the disease [5]. In addition, Taylor et al. [5] emphasized that the clinical presentation is diverse, the presence of pelvic lesions is heterogeneous, and the manifestations of the disease outside the female reproductive tract remain poorly understood. They concluded that endometriosis is a systemic disease and not one that predominantly affects the pelvis [5]. Although this criticism is justified in many respects, we really lack an understanding of how very small ectopic lesions can ultimately cause systemic disease in many patients. We propose including the uterus in these considerations, as the suppression of menstrual bleeding with contraceptives and hysterectomies with or without laparoscopy have cured endometriosis with low reoperation rates [6,7,8,9].

Although the Sampson hypothesis of retrograde menstruation [10] provides a reasonable model for ectopic endometrial tissue [11], it is still unclear why only 0.7–8.6% of women in the general population develop endometriosis [12]. Thus, several additional factors, such as inflammation, oxidative stress, the disturbance of the peritoneal barrier, and genetic/epigenetic changes, have been put forward to explain this discrepancy [13,14,15].

Studies have shown that the eutopic endometrium without and with endometriosis differs, suggesting that the onset of endometriosis may be in the endometrium [16,17]. Similar to Paget’s concept of “seed and soil” in tumorigenesis [17,18], endometriosis is also thought to be triggered by altered primary cells [16,17], even if these are not as extremely degenerated as tumor cells. The “seed” refers to tumor cells with metastatic potential, and the “soil” is any host tissue that provides a suitable environment for the seed to grow [17,18]. Thus, metastasis occurs only when the seed and the soil are compatible and preferentially rather than randomly in certain organs and not in others [17,18]. For endometriosis, this would mean that after retrograde menstruation [10], the pelvic and extra-pelvic tissues must be fertile ground for endometrial cells in order for endometriotic lesions to manifest.

The “seed and soil” concept is based on the following steps [17,18,19]: (1) the acquisition of cell alterations to the primary tissue (mutations, etc.), (2) the frequent loss of epithelial cell–cell contacts, (3) the acquisition of mesenchymal-like properties by epithelial–mesenchymal transition (EMT) resulting in a more motile phenotype, (4) the release of altered cells from the primary tissue due to the degradation of the extracellular matrix (ECM), (5) the evasion of the immune system, (6) migration and adhesion to host tissues, (7) the degradation of host tissue (ECM, etc.), (8) the invasion of the host tissue, and (9) the growth of the metastasis (cell proliferation, angiogenesis, etc.). With respect to endometriosis, we compare the initial steps of the “seed and soil” hypothesis because they are the prerequisite for cancer initiation and possibly are similar to endometriosis initiation. This is important because, in recent years, several cancer-driving mutations, such as KRAS, have also been found in endometrial and endometriotic epithelial cells, which might be linked to the development of pelvic endometriosis [20]. However, in a recent review, it was argued that aberrations in the eutopic endometrium are secondary to the establishment of ectopic foci [21].

What about another important aspect of cancer, the epithelial cell–cell contacts, the loss of which can lead to EMT? The analysis of epithelial cell–cell contacts, especially the claudins, revealed controversial data [22,23,24,25,26]; however, the endometrial epithelial phenotype is retained in the endometriotic ectopic lesions [1,27]. Recently, we suggested that only partial EMT without the loss of the epithelial phenotype might contribute to endometriosis [28].

In general, EMT is involved in wound healing, fibrosis, tissue regeneration, inflammation, and cancer metastasis [29,30,31] and is classified as follows: (1) type I EMT during embryonic development, (2) type II EMT during wound healing and tissue regeneration, and (3) type III EMT associated with cancer [30]. The gradual remodeling of epithelial cell architecture is a multistep process characterized by the first EMT hallmark: the loss of epithelial markers, leading to the disruption of cell–cell contacts, the remodeling of the cytoskeleton, and loss of apical–basal polarity. This is followed by the second hallmark of EMT, namely the acquisition of mesenchymal markers [29,30,31,32,33]. The cellular changes often lead to a mesenchymal phenotype with a spindle-like cell shape, increased cell migration, invasion, and cell survival (resistance to anoikis) [33,34]. Despite these significant changes, only a few transcription factors (TFs) or master regulators of EMT are involved. These include the Snail family proteins Snail1 (Snail), Snail2 (Slug), the Zinc finger E-box binding (Zeb) homeobox family proteins Zeb1 and Zeb2, and the TWIST family proteins Twist1 and Twist2 [35].

The first evidence of EMT was described for pelvic endometriosis by immunohistochemistry with EMT markers, such as cytokeratin, E/N-cadherin, vimentin, and S100A4 [36]. After attachment to the peritoneum, the reverse process called mesenchymal–epithelial transition (MET) was postulated to occur in peritoneal and deep infiltrating endometriosis [36]. Similarly, a decreased expression of cytokeratin in ectopic compared to eutopic endometrium was found [37,38]; however, we demonstrated a stable expression of keratin 18, 19 and mucin-1 in eutopic and ectopic epithelial cells without any loss of the epithelial phenotype [27]. It needs to be emphasized again that ectopic endometriotic lesions still consist of epithelial glands surrounded by stromal cells without an apparent mesenchymal phenotype of the epithelial cells [1,27].

In this study, we compared for the first time in depth the “seed and soil” hypothesis with the pathogenesis of endometriosis. Special attention was paid to the changes in the seed and the initial phase. Therefore, we re-analyzed mRNA/cDNA arrays to take a closer look at the key differences between endometrium without and with endometriosis and between eutopic and ectopic endometrium. In particular, the mRNA expression of cell–cell contacts and EMT-associated genes were analyzed.

## 2. Materials and Methods

We followed the Preferred Reporting Items for Systematic Review and Meta-Analyses Literature Search Extensions (PRISMA-S) guidelines [39] for this systematic review (Table S1). The study is registered (INPLASY202460009).

### 2.1. Search Strategy and Eligibility Criteria

We performed a systematic search in PubMed from 1990 up to 1st April 2023. We used the keywords “array”, “mRNA expression”, and “cDNA library” in conjunction with “endometriosis”, “eutopic endometrium”, “ectopic endometrium” (“endometrioma, peritoneal endometriosis, deep infiltrating endometriosis”) and “seed and soil”. All human studies reporting original data concerning mRNA expression, cDNA array, eutopic/ectopic endometrium, and endometriosis, as well as related studies, were included in this review. Studies not published in peer-reviewed journals were excluded. Only studies published in English were included.

### 2.2. Study Selection

**Figure 1:**
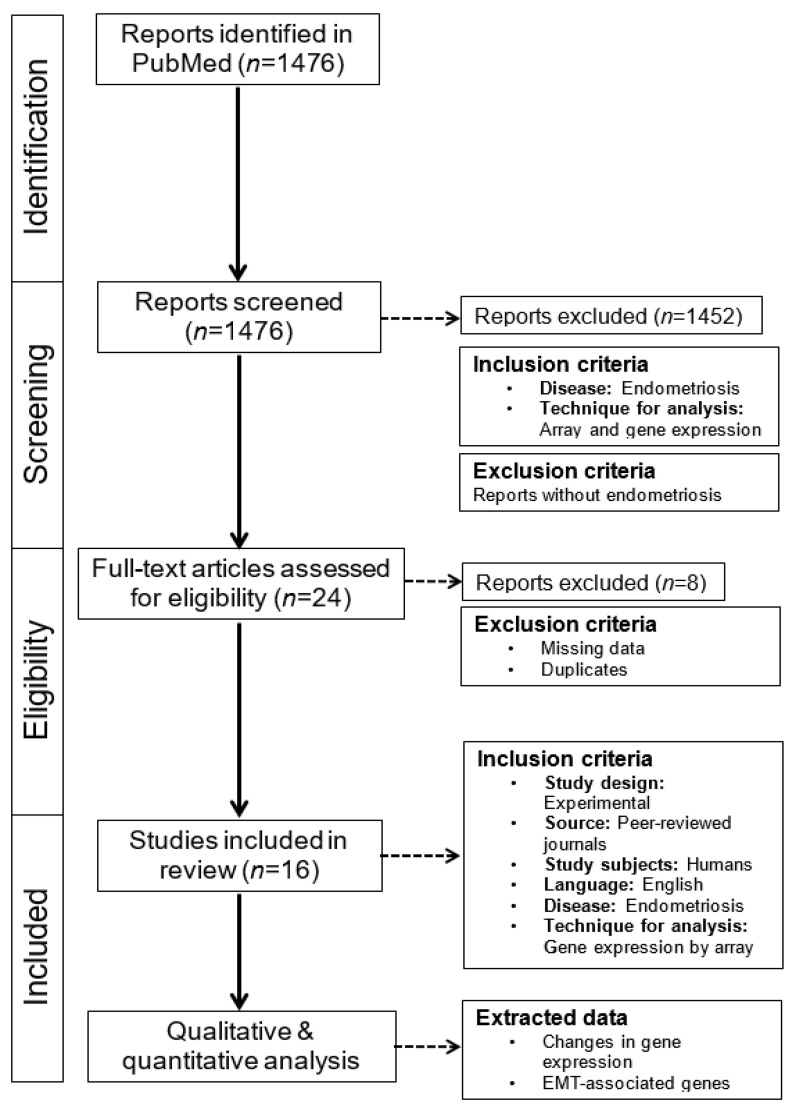
PRISMA flowchart of the literature search and data selection. This systematic retrospective review is based on literature research conducted in PubMed. The main focus was on mRNA/cDNA array analysis, EMT, and endometriosis in the eutopic and ectopic endometrium. These reports were carefully read, and data were extracted.

The results of the first search were summarized, and duplicates were deleted. The screening of titles and abstracts was performed independently by two authors (MAR and LK). The full texts were read and reviewed independently by the authors (MAR and LK), and each study was evaluated for inclusion using the specified eligibility criteria. Any disagreements were resolved through discussion between the authors until a consensus was reached. Additional studies were identified by screening the reference lists of the included studies. A summary of the work chart PRISMA is given in Figure 1.

### 2.3. Data Extraction and Synthesis

Data were extracted independently by MAR and LK. We looked for the following keywords: E/N-cadherin (CDH1/2), Snail1, Slug (also known as Snail2), Twist, claudin(s), Zeb1/2, integrin(s), keratin(s), and transforming growth factor-betas (TGF-βs).

Data included the title, author, journal, year of publication, population studied, phase of menstrual cycle, endometrium with and without endometriosis, outcomes, and results (Table 1). The results were then sorted thematically, and the authors determined the number of altered probes/genes compared to the total number in the final list of included studies. Claudins and other EMT-associated genes were examined in more detail. This final list was discussed until a consensus was reached among the authors. A meta-analysis was not possible in this review due to the heterogeneity of the methodology and the results of the papers included in the study.

## 3. Results

A total of 16 studies were included in the analysis (Table 1), 5 of which compared eutopic endometrium without endometriosis bit with eutopic endometrium and endometriosis, and 11 studies compared eutopic endometrium with endometriosis and ectopic endometrium (Table 1). Most manuscripts provided the age of the patients (11/16), the cycle phases (15/16), the use of OCs (16/16), the cell types/tissue isolated (16/16), staging (10/16), and the threshold used for up- and down-regulated genes (15/16). Differences in gene expression between the cycle phases (proliferative vs. secretory) were reported in 7/8 studies. Out of 10 studies, different stagings were reported in only two studies [48,53], and a different gene expression pattern between ovarian and non-ovarian ectopic endometrial lesions [48] or between stages 3 and 4 was described [53]. In one study with isolated EECs and ESCs, no different expression pattern between both cell types was found [47].

The comparison of eutopic endometrium without endometriosis and with eutopic endometrium and endometriosis revealed a total of 1195 out of 129,937 (0.92%) genes or samples with altered mRNA expression (Table 2). A slight regulation of claudin-3, claudin-6, claudin-10, and claudin-14 ranging from 0.59 up to 2.3 was described in only 2 of 5 manuscripts (Table 3) [40,42]. Additionally, TGF-β3 expression was also found to be increased in the eutopic endometrium of endometriosis patients compared to eutopic endometrium without endometriosis (Table 3) [40,42]. None of the other EMT-associated genes was found to be regulated in more than one study.

In 11 studies, the comparison of the eutopic endometrium with the ectopic endometrium showed a high percentage (4.74%, 15,234/321,149) of genes/samples with an altered mRNA expression (Table 4). In total, the altered expression of genes/samples in the ectopic endometrium compared to the eutopic endometrium (4.74%) was ~5× higher compared to the eutopic endometrium (0.92%, Table 2 and Table 4).

The comparison of eutopic with ectopic endometrium revealed the increased expression of claudin-1, -5, -6, -9, -11, -15, and -17 (Table 5). Remarkably, claudin-11 showed the highest scores of increased ectopic gene expression, ranging from 54.5 up to 100 in three different studies (Table 5). Claudin-2, -3, -4, -7, -10, and -22 demonstrated a slight-to-modest decreased expression in the ectopic endometrium compared to the eutopic endometrium (Table 5). Furthermore, TGF-β3 expression is also increased in ectopic endometrium compared to eutopic endometrium (Table 5).

## 4. Discussion

### 4.1. Comparison of mRNA Expression between Eutopic Endometrium with and without Endometriosis

In this study, a comparison of eutopic endometrium without and with endometriosis revealed only a small difference (0.92%) in mRNA expression. Accordingly, there were very few changes in the EMT-associated genes. Only four claudins (Cld3, 6, 10, 14) and TGF-β3 were abnormally expressed, whereas the expression of the other EMT-associated genes was not mentioned in more than one study.

Our observation of only very few differences in eutopic endometrium without and with endometriosis is supported by a recent meta-analysis, which did not show a single differently expressed gene in the mid-secretory phase [56]. Remarkably, other studies about methylation patterns [57,58,59] and miRNAs [60] also reported similar results. The methylation pattern only revealed differences (0–0.002%) between the eutopic endometrium with and without endometriosis, while considerably greater methylation patterns (0.18–28.8%) were dissimilar between the eutopic and ectopic endometrium [57,58,59]. Most of the different methylation patterns have been attributed to the cycle phases [59]. Similarly, the microRNA differences between the eutopic endometrium with and without endometriosis were comparatively low with 15/1105 (=1.36%) but noticeably more frequent between the eutopic and ectopic endometrium 156/1105 (=14.1%) [60]. Another study using subtractive hybridization found the same gene expression profile between eutopic endometrium with and without endometriosis [61].

Interestingly, in one array study, no reduction in epithelial and no gain in stromal cell characteristics in eutopic endometrium without and with endometriosis was found [55]. All the other studies are consistent with our observation that changes in mRNA expression in eutopic endometrium without and with endometriosis are minimal and that the expression differences between eutopic and ectopic endometrium are clearly larger.

Based on our findings, we can cautiously assume that the initiation of endometriosis seems not to be dependent on changes in the mRNA expression profile of the endometrial cells, or if it is, it depends only on very minor changes. This is a clear contrast to tumor cells, which usually acquire mutations that are the hallmark of cancers and are termed genome instability and mutation [62]. Even the recent findings of KRAS mutations in the endometrium of women with endometriosis do not change the picture, as all these mutations were found after the women already had endometriosis [21] and were also found in the normal endometrium [63]. A recent review such as our study, therefore, also questions the relevance of endometrial changes in endometriosis as a starting point for the pathogenesis of endometriosis [21].

Since the first step, or initiation, of the “seed and soil” hypothesis clearly differs from the onset of endometriosis, what about the following steps of the “seed and soil” hypothesis in relation to endometriosis (Table 6)?

### 4.2. Cell–Cell Contacts in the Eutopic Endometrium with and without Endometriosis

In this study, we identified only four claudins (claudin-3, -6, -10, and -14) with altered expression in eutopic endometrium with and without endometriosis. Our immunohistochemical analyses did not reveal any differences in the localization of claudins when comparing eutopic endometrium with or without endometriosis, neither for claudin-2, -3, -7, -10, or -11 [24,25,26]. Although not all endometrial epithelial cell–cell contacts has been studied in detail thus far, we hypothesize that it is highly likely that very few, if any, differences will be found. Thus, the loss of epithelial cell–cell contacts, which is the first hallmark of EMT, does not occur in eutopic endometrium with and without endometriosis. Except for one study, no down-regulation of E-cadherin (CDH1) was described [50], and no regulation of N-cadherin (CDH2) was found in any array study. In contrast, in endometrial cancer cells, E-cadherin was down-regulated, and N-cadherin was up-regulated, resulting in EMT [64]. Consequently, the epithelial phenotype of the endometrial epithelial cells is still clearly preserved in the eutopic endometrium without and with endometriosis [1,27]. This observation also shows a clear difference between endometrial epithelial cells and tumor cells with respect to the second step of the “seed and soil” hypothesis (Table 6).

### 4.3. EMT in the Eutopic Endometrium with and without Endometriosis

After the progressive loss of much epithelial cell–cell contacts for the tumor cells, the expression of mesenchymal markers increases during the process of EMT [65], although EMT is not observable in some tumors [66,67]. Our comparison of eutopic endometrium without and with endometriosis did not yield a single difference in the mRNA expression levels of EMT modulators, such as Snail1, Snail2, Zeb1, Zeb2, Twist1, and Twist2. Therefore, we propose that EMT does not play a role in the initiation of endometriosis. As summarized in our previous review on EMT, most of the studies on EMT have examined the acquisition of mesenchymal markers but not the loss of epithelial markers, particularly for cell–cell contacts such as the claudins [28]. The small increase in the mesenchymal gene expression of endometrial epithelial cells does not allow the assumption of a transition to mesenchymal cells; at most, the conclusion of a partial EMT without the loss of the epithelial cell characteristics can be drawn [28]. Although a recent bioinformatic analysis of three microarray datasets emphasized the importance of EMT in the development of endometriosis due to down-regulation of E-cadherin (CDH1) [68], another study ruled out EMT in the endometrium, as only the endometrial epithelial cells but not the endometrial stromal cells, showed DNA alterations/mutations [27]. In the case of EMT, the observed mutations in the endometrial epithelial cells should also have been found in the endometrial stromal cells, which was not the case [27].

### 4.4. Comparison of mRNA Expression between Eutopic and Ectopic Endometrium

Our analysis of gene expressions between the eutopic and ectopic endometrium showed about ~5 times more differences (4.74%) compared to that of eutopic endometrium with and without endometriosis (0.92%), although one study using cell picking revealed no differences in gene expression between eutopic and ectopic endometrium [47]. We can, thus, conclude with a high degree of certainty that most of the differences in gene expression did not occur before but after the implantation of endometrial cells. We assume that these changes are caused by the interactions of ectopic endometrial cells with the environment, as already postulated by other authors [69,70]. These interactions are also the cause of the alteration of the host tissue by the endometriotic implants, often resulting in fibrosis [71]. Remarkably, only one other group reached the same conclusions as we did. They stated that the differences between the eutopic endometrium and the ectopic lesions, as well as between the ectopic lesions, were a direct result of the different environments ((peritoneal fluid (PF) and intraovarian environment)) compared to the intrauterine environment [59].

Although more claudins (*n* = 15) and TGF-β3 were abnormally expressed in the ectopic compared to the eutopic endometrium with and without endometriosis, EMT-regulating transcription factors such as Zeb1/1 and Snail1/2 were not described in more than one study. Of note, the mRNA expression of claudin-11 was often strongly increased in the ectopic endometrium compared to the eutopic endometrium. Overall, ovarian endometriosis was examined frequently, but only one study examined ovarian and non-ovarian endometriosis in more detail and found a significant difference [48]. A recent array study demonstrated convincingly that the gene expression in ovarian endometriosis is significantly different from peritoneal as well as from deep-infiltrating endometriosis [72].

### 4.5. EMT in the Eutopic Endometrium Compared to the Ectopic Endometrium

Only one array of endometriotic lesions showed reduced epithelial cell characteristics and a gain of stromal cell characteristics in contrast to eutopic endometrium [55]. Therefore, these observations support our hypothesis of a partial EMT without loss of the epithelial phenotype, which we put forward from the immunohistochemical analysis of EMT-associated proteins of the eutopic and ectopic endometrium [28]. Our conclusions are further corroborated by the results of the DNA sequencing of endometrial epithelial and stromal cells, in which no shared mutations in both cell types were found [21,73,74,75]. Similarly, a mouse model for endometriosis showed no EMT (no change in cytokeratin and E-cadherin levels) but instead showed proliferation and inflammation to be responsible for endometriosis [76]. In contrast, EMT is one of the most important functions of claudin proteins in cancer progression [77], and although EMT is important, it is not classified as a hallmark of cancer metastasis [78].

Although we cannot completely rule out the possibility that circulating endometrial cells (CECs) may have been altered by EMT prior to implantation, there has been no study to date showing the alterations of CECs by EMT. Remarkably, endometrial tissue fragments from endometriosis and control patients did not differ in their implantation potential on chorionic allantois membranes (CAMs) in vitro [78]. The authors suggest that implantation is possibly determined by external factors regulating influences on the endometrial implants [79]. Similarly, Nap et al. [80] showed that the integrity of endometrial tissue architecture determined the success of the implantation of the human endometrium into CAM ectopic locations in vitro.

Our analysis of EMT-associated genes showed altered expression patterns only for claudin-3, -6, -10, and -14 and TGF-β3 in eutopic endometrium without and with endometriosis while a comparison between the eutopic and ectopic endometrium revealed an aberrant gene expression of many claudins, claudin-1 up to claudin-11, and claudin-15, -17, and -22. The highest increase in expression was reported for claudin-11 [50,52,55]; however, this was without any supporting protein data. In contrast, we observed only a moderately decreased abundance of claudin-11 in ovarian endometriosis compared to eutopic endometrium [24]. Claudin-11 was localized mainly in the apicolateral junctions in nearly all glandular epithelial cells of the eutopic endometrium. Interestingly, the deregulation of claudin-11 localization to basal or basolateral localization in ovarian, peritoneal, and deep-infiltrating endometriosis was observed [24]. The silencing of claudin-11 decreased the invasiveness of endometriotic epithelial 12Z cells only slightly but significantly increased invasiveness in endometriotic epithelial 49Z cells [24].

In contrast to two reports that described an impaired expression of claudin-3 in endometriosis [22,23], we recently found an unchanged protein localization in the eutopic endometrium both with and without endometriosis and also in the ectopic endometrium [25], which might be due to the different fixation protocols used. Similarly, we found a high abundance (~98%) of claudin-10 in nearly all endometrial and endometriotic glands but no differences in the claudin-10-positive endometrial glands between cases with and without endometriosis [26]. A significantly higher expression of claudin-10 was detected in the ectopic endometrium of deep-infiltrating and ovarian endometriosis [26]. Interestingly, a shift in claudin-10 from a predominant apical localization in the eutopic endometrium to a more pronounced basal/cytoplasmic localization in the ectopic endometria of all three endometriotic entities (ovarian, peritoneal, deep infiltrating) was observed [26]. Of note, despite the impaired endometriotic localization of claudin-10, the epithelial phenotype was retained in all glands [26].

A decreased expression of claudin-7 was observed in ectopic compared to eutopic endometrium in the array studies [52,55] as well as with immunohistochemistry [22]. Claudin-7 was identified primarily at the basolateral junctions of the glandular epithelial cells in the eutopic endometrium as well as in the ectopic lesions in nearly all glands and cysts [24]. The quantification of claudin-7 localization showed a slight increase in peritoneal and deep-infiltrating endometriosis compared to the eutopic endometrium [24].

None of the other claudins have been analyzed in more depth in endometriosis to date. In contrast, a recent review of claudin expression in endometrial cancer described an up-regulation of claudin-1-4, -6 and -9 and a down-regulation for claudin-7 only [81].

Beyond the three isoforms of the TGF-βs, the TGF-β1-3 expression of TGF-β3 mRNA was increased in eutopic endometrium with endometriosis compared to those without endometriosis [40,42]. Similarly, TGF-β3 gene expression was also higher in the ectopic compared to eutopic endometrium [49,52]. TGF-β1 showed the highest expression compared to TGF-β2 and TGF-β3 in the human endometrium [82]. TGF-β3, preferentially expressed in the stroma, was increased at menstruation and remained high during the proliferative phase. In contrast, TGF-β1 was elevated in the PF of women with endometriosis compared to those without the disease, while TGF-β3 was not altered [83]. However, higher PF and serum levels of TGF-β1, -β2, and -β3 were observed in women with endometriosis compared to the controls [84].

### 4.6. Is the Seed and Soil Concept also Applicable for Endometriosis?

Our comparison of the first steps of the initiation of endometriosis with the seed and soil hypothesis of cancer reveals that although the sequence of events is the same, the individual steps differ significantly (Table 6). Tumor metastases represent distinct subsets of cells that leave the primary tumor and are behaviorally, genetically, and biochemically distinct from the cells remaining at the site of the primary tumor [78]. The escape of neoplastic cells through a basement membrane is a hallmark of malignancy/metastasis but not EMT [78]. In clear contrast to tumor cells, the endometrial epithelial and stromal cells show only minor, if not negligible alterations. Endometrial cells, to become endometriotic, neither acquire additional properties for tissue breakdown, which happens regularly during menstruation nor do they lose their cell–cell contacts and do not undergo EMT (Table 6). Another review reached similar conclusions and stated that molecular aberrations as a sole or a necessary determinant for endometriosis remains to be proven [21].

In the array studies presented, the changes in the soil, such as the formation of a pro-endometriotic niche [84], have not been investigated so far, but the changes in the seed have been recorded. However, it is important to note that there is a lot of evidence [85,86], though as of yet no unequivocal proof, of the formation of a pro-endometriotic niche in endometriosis. Furthermore, another fundamental difference between cancer metastases and endometriotic lesions is that in endometriosis, the endometrial stroma usually migrates along with the epithelium and then integrates into the stroma of the host tissue (Table 6). In tumor metastases, altered epithelial cells usually integrate into the host stroma, but again, it must be emphasized that the endometrial cells are still recognizable as such; they do not lose their cell–cell contacts, and if a partial EMT takes place, the epithelial phenotype is not lost [1,28]. Furthermore, the tumor metastases can significantly damage the surrounding host tissue [78], which, with few exceptions, is in clear contrast to endometriotic implants, which are very limited in size.

## 5. Strength and Limitations

This is the first study comparing the initiation of the pathogenesis of endometriosis with the “seed and soil” hypothesis, and to evaluate the similarities and differences in gene expression between the eutopic endometrium with and without endometriosis with special emphasis on EMT-associated genes. One limitation lies in the fact that the total number of samples is higher than the real number of genes. However, the analysis was less about absolutes and more about relative values. Another limitation is that up until now, the (pre)-endometriotic niche (=soil) has not been analyzed by any array of studies.

## 6. Conclusions

The results of this study clearly show very little differences in gene expression between the eutopic endometrium with and without endometriosis. Furthermore, it must be noted that these differences were identified after and not before the onset of endometriosis. Therefore, like the currently known mutations, these changes in the mRNA expression pattern cannot be regarded as the cause of endometriosis but, at present, only as an epiphenomenon. In contrast, the differences between the eutopic and ectopic endometrium are much more significant, and it is highly likely that they are the consequence of the interaction between the ectopic endometrium and the surrounding microenvironment. We suggest that most, if not all, changes happen after and not before implantation. Remarkably, there were also few differences in the expression of EMT-associated genes with the complete absence of master genes. Therefore, we assume that there is at most only a partial EMT, with no loss of the epithelial phenotype, and that EMT plays only a minor, if not negligible role, in the initiation of endometriosis. The comparison of endometriosis with the “seed and soil” hypothesis of tumorigenesis showed a similar sequence of events in the initiation phase, but the individual steps were considerably different.

## Figures and Tables

**Table 1:** Characteristics of patients, tissues and array threshold.

Uteri	Ref.	Age	Cycle Phase	OC	Cell Type	Staging	Threshold
	[40]	28–39	S, regular	No	Tissue	rAFS2/3 (*n* = 8)	2, 0.5
	[41]	19–48	P, S	Yes	Tissue	n.st.	1.5, 0.667
	[42]	22–44	P, S, regular, infertile (some)	No	Tissue	rAFS3/4	1.5, 0.667
	[43]	n.st.	S, regular	No	Tissue	rAFS1/2 (*n* = 4)	1.75, 0.57
						rAFS3/4 (*n* = 4)	
	[44]	24–43	M, P, regular (*n* = 35)	No	Tissue	ASRM1/2 (*n* = 16)	2, 0.5
			irregular (*n* = 10)			ASRM3/4 (*n* = 15)	
**EM**	**Ref.**	**Age**	**Cycle Phase**	**OC**		**Staging**	**Threshold**
	[45]	n.st.	S	No	Tissue	n.st.	n.st.
	[46]	23–44	P, S	No	EEC	n.st.	2, 0.5
	[47]	n.st.	P, S, Regular	No	EEC, ESC	n.st.	3, 0.33
	[48]	25–44	P, S	No	EEC	rAFS2 (*n* = 1)	1.5, 0.667
						rAFS3 (*n* = 5)	
						rAFS4 (*n* = 6)	
	[49]	22–40	P	No	Tissue	n.st.	2, 0.5
	[50]	28–45	P, S, infertile (10/11)	No	Tissue	rAFS2/3 (*n* = 5)	2, 0.5
						rAFS4 (*n* = 6)	
	[51]	n.st.	P, infertile	No	Tissue	rAFS3/4 (*n* = 14)	2, 0.5
	[52]	n.st.	n.st.	No	Tissue	Stage 4 (*n* = 6)	2, 0.5
	[53]	24–45	P, S, regular	No	Tissue	ASRM3 (*n* = 8)	3, 0.33
						ASRM4 (*n* = 10)	
	[54]	24–46	S	No	Tissue	n.st.	2, 0.5
	[55]	21–52	P, S	No	Tissue	rAFS1/2 (*n* = 17)	5, 0.2
						rAFS3/4 (*n* = 10)	

OC, oral contraceptives; Ref., reference; n.st., not stated; EM, endometriosis; P, proliferative, S, secretory; M, menstrual; EEC, endometrial epithelial cells; and ESC, endometrial stromal cells.

**Table 2:** Genome-wide analysis of eutopic endometrium with and without endometriosis.

Ref.	Endometrium Healthy	Endometrium with Endometriosis	Altered Gene Expression	%	All Lesions
[40]	*n* = 7	*n* = 8	206/12,686	1.6	n.d.
[41]	*n* = 41	*n* = 43	95/12,651	0.8	*n* = 19
[42]	*n* = 16	*n* = 21	885/54,600	1.62	n.d.
[43]	*n* = 6	*n* = 10	9/22,000	0.04	n.d.
[44]	*n* = 18	*n* = 31	0/28,000	0	n.d.
Sum	88	113	1195/129,937	0.92	(mean)

Here, 206/12,686 means that 206 out of 12,686 samples showed an altered gene expression, Ref., reference. n.d., not done.

**Table 3:** Up- or down-regulation of EMT-associated genes in eutopic endometrium without versus with endometriosis.

Genes	Up-Regulation	Down-Regulation	References
Claudin-6	1.54	-	[42]
Claudin-10	2.3	-	[40]
Claudin-3	-	0.59	[42]
Claudin-14	-	0.65	[42]
TGF-β	100	-	[40]
TGF-β3	3.14	-	[42]

**Table 4:** Genome-wide analysis of eutopic and ectopic endometrium.

EM Healthy	EM with Endometriosis	Sum Lesions (Paired)	Altered Gene Expression	OMA	PE	DIE	Ref.
n.d.	*n* = 3	*n* = 3	8/4133 (0.2%)	*n* = 3	n.d.	n.d.	[45]
n.d.	*n* = 23	*n* = 23	1413/23,040 (6.1%)	*n* = 23	n.d.	n.d.	[46]
n.d.	*n* = 12	*n* = 12	0/1176 (0%)	n.d.	n.d.	*n* = 12	[47]
n.d.	*n* = 12	*n* = 25	904/9600 (9.4%)	*n* = 6	*n* = 5	*n* = 1	[48]
			(904/4684 * = 19.3%)				
*n* = 5 (not for array)	*n* = 5	*n* = 5	13/1176 (1.1%)	*n* = 5	n.d.	n.d.	[49]
			(12/940 * = 1.38 *)				
n.d.	*n* = 10	*n* = 10	1146/53,000 (2.16%)	yes	yes	not sp.	[50]
n.d.	*n* = 4	*n* = 4	36/44,928 (0.08%)	*n* = 4	n.d.	n.d.	[51]
n.d.	*n* = 6	*n* = 6	5600/53,000 (10.6%)	*n* = 6	n.d.	n.d.	[52]
n.d.	*n* = 18	*n* = 18	847/29,421 (2.88%)	*n* = 18	n.d.	n.d.	[53]
n.d.	*n* = 6	*n* = 6	1366/47,000 (2.9%)	*n* = 6	n.d.	n.d.	[54]
n.d.	*n* = 17	*n* = 18	3901/54,675 (7.1%)	n.d.	*n* = 18	n.d.	[55]
Sum			15,234/321,149 (4.74%)				
			15,234/314,821 * (4.84% *)				

Here, 8/4133 means that 8 out of 4133 samples showed an altered mRNA expression. The percentage of altered samples is given in brackets. * In two studies, the numbers of mRNA changes per gene were included. The calculation change per gene only slightly increased with the rate of change (4.74% vs. 4.84%). EM, endometrium; Ref., references; OMA, endometrioma; PE, peritoneal endometriosis; DIE, deep infiltrating endometriosis; n.d., not done; and not sp., not specified.

**Table 5:** Up- or down-regulation of EMT-associated genes in eutopic endometrium compared to ectopic endometrium.

Genes	Up-Regulation	Down-Regulation	References
Claudin-1	6.64	-	[55]
Claudin-1	0.87–2.85	-	[53]
Claudin-5	4.31	-	[55]
Claudin-5	7.46	-	[53]
Claudin-6	1.05	-	[53]
Claudin-9	2.16	-	[53]
Claudin-11	54.05	-	[55]
Claudin-11	69.3	-	[53]
Claudin-11	100	-	[50]
Claudin-15	1.31–2.07	-	[53]
Claudin-17	1.25	-	[53]
Claudin-2	-	0.45–0.55	[53]
Claudin-3	-	0.14	[55]
Claudin-3	-	0.06	[53]
Claudin-3	-	0.58	[50]
Claudin-4	-	0.11	[55]
Claudin-4	-	0.1	[53]
Claudin-7	-	0.19	[55]
Claudin-7	-	0.12	[53]
Claudin-8	-	0.28	[53]
Claudin-10	-	0.17	[53]
Claudin-22	-	0.17	[53]
TGF-β3	4.86	-	[53]
TGF-β3	0.9–1.7	-	[49]

**Table 6:** Comparison of the initial steps of epithelial cancers and endometriosis (epithelial cells).

Steps	Cancer (Epithelial)	Endometriosis (Epithelial)
Alterations in primary tissue	Many	Some somatic mutations
2. Loss of cell–cell contact	Loss	Negligible loss
3. EMT	EMT in most cancers	Partial EMT
4. Epithelial phenotype	Changes/loss	Negligible loss
5. Cell release (ECM degradation etc.)	Necessary	During menstruation
6. Stromal cells	None	Co-migration with EECs

EEC, endometrial epithelial cells.

## Data Availability

The data presented in this study are taken all from the references as indicated. The new data we have generated are all summarized in the Tables in the manuscript.

## Supplementary Materials

The following are available online at https://www.mdpi.com/article/10.3390/biomedicines12061276/s1, Table S1: PRISMA-S checklist.
