# Supplementary material for: The Different Gene Expression Profile in the Eutopic and Ectopic Endometrium Sheds New Light on the Endometrial Seed in Endometriosis
Source: Biomedicines. 2024 Jun 8;12(6):1276. doi: 10.3390/biomedicines12061276 (PMC11201009; doi:10.3390/biomedicines12061276)
Supplement: Supplementary file 1 [file biomedicines-12-01276-s001.zip › biomedicines-3005716-supplementary.pdf]

Table S1 PRISMA-S checklist

Item 1	Medline (PubMed)
Item 2	N/A
Item 3	Not done
Item 4	Google
Item 5	Reference lists of relevant articles were manually searched to identify additional studies.
Item 6	We contacted authors for more information but it was in vain
Item 7	We checked PubMed's 'similar articles' and 'cited by' features.
Item 8	We used the keywords “array”, “mRNA expression”, “cDNA library” in all combinations with “endometriosis”, “eutopic endometrium”, “ectopic endometrium” (“endometrioma, peritoneal endometriosis, deep infiltrating endometriosis”) or “seed and soil”.
Item 9	The review was limited to the English language and peer-reviewed journals. Without peer review studies are worthless.
Item 10	We performed a systematic search in PubMed from 1990 up to 1st April 2023
Item 11	N/A
Item 12	The search was repeated annually since 2020
Item 13	The last search was 1st April 2023
Item 14	Screening of titles and abstracts was performed by two authors independently. The full texts were read and reviewed independently and each study was evaluated for inclusion using the specified eligibility criteria. Any disagreements were resolved through discussion between the authors until a consensus was reached
Item 15	1476 hits were generated
Item 16	Deduplication was done manually

Guidelines for PRISMA-S were used as published [39]
